# Asian wild rice is a hybrid swarm with extensive gene flow and feralization from domesticated rice

**DOI:** 10.1101/gr.204800.116

**Published:** 2017-06

**Authors:** Hongru Wang, Filipe G. Vieira, Jacob E. Crawford, Chengcai Chu, Rasmus Nielsen

**Affiliations:** 1 State Key Laboratory of Plant Genomics, National Center for Plant Gene Research (Beijing), Institute of Genetics and Developmental Biology, Chinese Academy of Sciences, Beijing 100101, China;; 2 College of Life Sciences, University of Chinese Academy of Sciences, Beijing 100101, China;; 3 Centre for GeoGenetics, University of Copenhagen, 1350 Copenhagen, Denmark;; 4 Department of Integrative Biology, University of California, Berkeley, California 94720, USA

## Abstract

The domestication history of rice remains controversial, with multiple studies reaching different conclusions regarding its origin(s). These studies have generally assumed that populations of living wild rice, *O. rufipogon*, are descendants of the ancestral population that gave rise to domesticated rice, but relatively little attention has been paid to the origins and history of wild rice itself. Here, we investigate the genetic ancestry of wild rice by analyzing a diverse panel of rice genomes consisting of 203 domesticated and 435 wild rice accessions. We show that most modern wild rice is heavily admixed with domesticated rice through both pollen- and seed-mediated gene flow. In fact, much presumed wild rice may simply represent different stages of feralized domesticated rice. In line with this hypothesis, many presumed wild rice varieties show remnants of the effects of selective sweeps in previously identified domestication genes, as well as evidence of recent selection in flowering genes possibly associated with the feralization process. Furthermore, there is a distinct geographical pattern of gene flow from *aus*, *indica*, and *japonica* varieties into colocated wild rice. We also show that admixture from *aus* and *indica* is more recent than gene flow from *japonica*, possibly consistent with an earlier spread of *japonica* varieties. We argue that wild rice populations should be considered a hybrid swarm, connected to domesticated rice by continuous and extensive gene flow.

Asian cultivated rice is one of the most ancient and widely consumed staple food crops. Its domestication and cultivation contributed to the rise of agricultural civilization in Asia. Rice is believed to have been domesticated ∼9000 yr ago from one of its sympatric wild species, *O. rufipogon* (Oka 1988; Fuller et al. 2010). Molecular studies have identified multiple varietal groups in cultivated rice, including two major ones: *japonica* (*keng*) and *indica* (*hsien*) (Glaszmann 1987; Garris et al. 2005; Sweeney et al. 2007). *Indica* and *japonica* are highly differentiated and partially reproductively isolated by a postzygotic barrier (Chang 2003). Despite numerous archaeological and genetic studies on the history of rice domestication, no consensus has been reached on the number of origins of different rice subgroups (Sang and Ge 2007; Huang et al. 2012b; Civáň et al. 2015). Some researchers argue for a single-origin model, which hypothesizes that rice domestication was a single event followed by a post-domestication diversification that created divergent subgroups. This model is supported by molecular research on the domestication genes, *sh4* (Li et al. 2006; Lin et al. 2007) and *PROG1* (Jin et al. 2008; Tan et al. 2008), which are responsible for two of the most critical domestication traits in rice, nonshattering grains and erect growth, respectively. It has been shown that different varietal groups of cultivated rice share identical sequences at these two domestication genes (Lin et al. 2007; Tan et al. 2008). Additionally, multiple studies that inferred the demographic histories of domesticated rice using independent data sets favor the single-origin model (Gao and Innan 2008; Molina et al. 2011). However, phylogenetic analyses using both nuclear and cytoplasmic DNA markers consistently show that *indica* and *japonica* are each associated with different subgroups of *O. rufipogon* (Cheng et al. 2003; Zhu and Ge 2005; Londo et al. 2006; Rakshit et al. 2007; Huang et al. 2012b; Civáň et al. 2015). Some have used these results to argue that rice domestication occurred more than once, and they attribute the sharing of key domestication loci to gene flow after domestication (Londo et al. 2006; Rakshit et al. 2007; Sang and Ge 2007) or independent selection from standing ancestral variation (Civáň et al. 2015).

Despite these seemingly conflicting viewpoints, it is well accepted that understanding the genetic variation of the primary gene pool, from which rice was domesticated, is critical in studying rice domestication (Vaughan et al. 2008). The primary gene pool is a concept used among plant breeders to define a set of species/subspecies comprised of three components: the cultivated species, its wild ancestor, and in many cases, its weedy counterparts (Harlan and DeWet 1971). Within this gene pool, hybridization occurs easily and hybrid swarms are occasionally formed as a result of crossing between the constituent components (Harlan 1992).

There has been extensive work on the population structure and genetic relatedness of different subgroups within the rice primary gene pool, but incongruent phylogenetic patterns have been observed. Wild rice has an annual ecotype, *O. nivara*, and its phylogenetic position with the perennial type is inconclusive (Lu et al. 2002; Londo et al. 2006); thus in this study, we will not separate it from *O. rufipogon*. Early molecular phylogenetic studies using isozymes identified two genetic groups of *O. rufipogon*, with closer genetic affinity to *indica* and *japonica*, respectively (Second 1982). Multiple other DNA studies also identified different genetic subgroups in wild rice populations associated with different domesticated rice subgroups. In addition, they also identified more ancestral genetic groups in wild rice population (Sun et al. 1996; Cheng et al. 2003; Zhu and Ge 2005; Londo et al. 2006). Using genome-wide markers from 48 sequence-tagged sites, Huang et al. (2012a) concluded that there were two distinct groups of wild rice, one genetically related to *indica*, and one without particular relatedness to any domesticated group. A whole-genome sequencing study (Huang et al. 2012b) categorized wild rice into three groups, two of which cluster with *japonica* and *indica*, respectively, in the phylogeny constructed with genome-wide SNP markers. Recently, a genotype-by-sequencing study on 286 diverse *O. rufipogon* species complex accessions identified six subpopulations and suggested that there was gene flow between *O. rufipogon* species complex and *O. sativa* (Kim et al. 2016). To account for the range of genetic variation within the rice primary gene pool, various modeling analyses were also performed (Caicedo et al. 2007; Zhu et al. 2007; Gao and Innan 2008; Molina et al. 2011). They consistently found that domesticated species had suffered severe bottlenecks and that models of nonindependent rice domestication provided better explanation for the pattern of genetic variation within the gene pool (Gao and Innan 2008; Molina et al. 2011). Also, field sampling studies in different regions observed ongoing gene flow among different components of the gene pool (Pusadee et al. 2013, 2016; for summary, see Oka 1988). Numerous concerns were raised regarding the conservation of genetic diversity in wild rice populations, because frequent gene flow from domesticated rice into wild rice populations could cause genetic erosion and diversity loss in wild rice (Oka 1988). It is also well recognized that gene flow between domesticated and wild rice populations is an important factor that might confound phylogenetic studies and demographic history inferences on the rice primary gene pool (Vaughan et al. 2008; Huang et al. 2012a). However, there is no study to date that estimates the amount of gene flow from domesticated rice into natural wild rice populations and/or determine the extent to which gene flow has shaped the genetic landscape of wild rice.

## Results

### Admixture analysis in the primary gene pool of Asian rice

To investigate population structure and admixture patterns in the primary gene pool of Asian rice, we combined whole-genome sequencing data from 203 cultivated rice varieties (Wang et al. 2016) and 435 accessions of *O. rufipogon* (Huang et al. 2012b). The cultivated rice accessions were collected from 71 countries and were systematically selected to be representative of rice diversity from more than 18,000 accessions in the USDA rice germplasm seed bank (Agrama et al. 2009). The wild rice samples were collected in situ in wild rice natural habitats (Supplemental Text S1) by scientists from the National Institute of Genetics in Japan (Morishima 2002).

We first estimated ancestry proportions for individuals using NGSadmix (Skotte et al. 2013), which implements a clustering method similar to the one in the popular program ADMIXTURE (Alexander et al. 2009), while incorporating uncertainty in the genotype calls inherent in next generation sequencing (NGS) data. We fit admixture models by varying the number of presumed ancestral populations (*K*) from 2 to 15 (Supplemental Figs. S1–S3). Generally, the results fit those found in previous studies and those expected from prior knowledge of rice population genetics (Supplemental Text S3). However, accessions of domesticated rice are identified to have a small amount (<5%) of wild rice ancestry, possibly reflecting introgression from wild rice, which was not observed in previous studies (Wang et al. 2016). In the most remarkable case, one domesticated rice accession (GSOR311586) was identified to be of 99% wild ancestry. We conducted field observations, which showed that this accession has shattering grains and black-hull seeds with long awns that are hallmark phenotypes of wild rice (Supplemental Fig. S6). PCR also confirmed that this accession contained a wild allele of *sh4*. It is very likely that this is, in fact, a wild rice accession that was misidentified as domesticated during germplasm collection.

**Figure 1.**
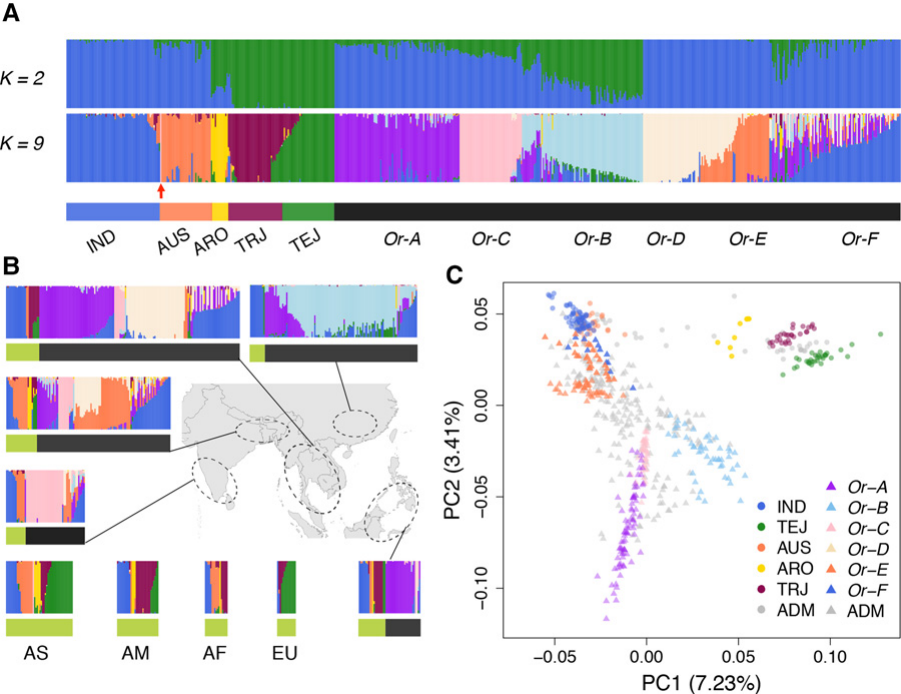
Population structure of the rice primary gene pool, including *O. sativa* and *O. rufipogon*. (*A*) Clustering using NGSadmix assuming *K* = 2 and *K* = 9. At *K* = 2, the samples are divided into *indica* and *japonica* components. At *K* = 9, five subgroups of domesticated rice are recovered, and four unique components of wild rice are identified. The color bars *beneath* the clusters denote the subgroup assignments. The red arrow points to the misidentified domesticated accession (GSOR311586), which was confirmed to have wild rice ancestry. The abbreviations of subgroups in cultivated rice are as follows: (ADM) admixture; (IND) *indica*; (AUS) *aus*; (ARO) *aromatic*; (TRJ) *tropical japonica*; (TEJ) *temperate japonica*. (*B*) Geographic distribution of rice samples. South and Southeast Asia, which are the major habitats for wild rice and also major rice cultivation areas, are shown on the map. The area was divided into five regions: (1) South Asia; (2) Ganges Basin; (3) Indochina Peninsula; (4) China; (5) Archipelago countries. The color code of the bar *beneath* the clustering plot indicates cultivated (green) and wild (black) rice. The abbreviations at the *bottom left* are as follows: (AS) Asia; all rice samples from Asia but not shown on the map are included in this category; (AM) America; (AF) Africa; (EU) Europe. (*C*) PCA of the combined population with wild (triangle) and cultivated (dot) rice samples. The abbreviation codes are the same as those in *A*.

In the wild rice population, however, we identified six subgroups (Fig. 1A), which we denote as *Or-A*, *Or-B*, *Or-C*, *Or-D*, *Or-E*, and *Or-F*, respectively, according to the order of emergence when increasing *K* in the admixture analyses (Fig. 1A; Supplemental Fig. S1). We also find good correspondence between subpopulations assigned here and previously described genetic subgroups (Huang et al. 2012b) based on phylogenetic analyses (Supplemental Table S1; Supplemental Fig. S7). Notably, a large proportion (42%) of wild rice individuals seems to be substantially admixed and thus could not be assigned to a single ancestry group, suggesting a complicated history of hybridization and differentiation among wild rice. Among the identified clusters, four components (*Or-A*, *Or-B*, *Or-C*, and *Or-D*) are unique to wild rice. The *Or-A* component is the first to emerge in wild rice when we increase *K* from 2 to 3. This component has a broad geographic distribution, with highest ancestry proportions concentrated in the oceanic regions and lower ancestry proportions in West India and Sri Lanka (Fig. 1B). *Or-B* emerged when five ancestral populations were included in the model. Geographically, *Or-B* is found almost exclusively in China, and it has been hypothesized that *Or-B* may represent the wild ancestor of both *indica* and *japonica* since this population harbors ancestral alleles at domestication-related loci shared by *indica* and *japonica* (Huang et al. 2012b). Adding one additional ancestral population to the model (*K* = 6) results in the emergence of *Or-C*, which is found mostly in South and Southeast Asia and comprises the majority of the wild rice genomes in the West India and Sri Lanka populations. *Or-D* is found almost exclusively in the Indochina Peninsula, Bangladesh, and East India (Fig. 1B). Intriguingly, for the last two subgroups (*Or-E* and *Or-F*), the major genetic components are shared with *aus* and *indica*, respectively. To further characterize the genetic relationships among subgroups in this gene pool, we carried out a principal component analysis (PCA) (Fig. 1C; Supplemental Fig. S8). In the PCA space constructed with the first two PCs, *japonica* forms an isolated cluster, whereas *indica* and wild rice form a separate, more diffuse cluster. *Or-E* and *Or-F* colocalize with *aus* and *indica* in the PCA plot. PC3 separates *indica* and *aus*, each forming a cluster. However, *Or-E* and *Or-F* still cluster with *aus* and *indica*, respectively, and the clustering pattern persists even at higher dimensions of the PCA space (Supplemental Fig. S8). This suggests a very high degree of genetic relatedness between wild rice subgroups *Or-E/Or-F* and the domesticated rice subgroups *aus*/*indica*, respectively.

### Gene flow between *O. rufipogon* and *O. sativa*

The exceptional genetic similarity between *Or-E*/*Or-F* and the corresponding domesticated subgroups revealed by PCA and admixture analyses can be explained by two possible hypotheses. First, *Or-E* and *Or-F* could be extant representatives of the ancestral source population used in the domestication process, and the genetic affinity with *aus* and *indica* could result from standing ancestral polymorphism segregating in these domesticated subgroups. Second, it could be caused by gene flow between domesticated rice and the corresponding wild subgroups. To test these hypotheses, we first conducted a correlation analysis between geographic distance and genetic distance in all *sativa–rufipogon* pairs. We find a highly significant correlation (ρ = 0.15, *P* < 2.2 × 10^−16^) (Supplemental Fig. S9), indicating that geographically close *sativa–rufipogon* sample pairs tend to be more genetically related than expected. One possible explanation for the correlation could be that the correlation is driven by shared ancestral polymorphism between two species, but this is only tenable when there are multiple geographic sites where rice was domesticated independently. Moreover, the correlation is also present within smaller regions, such as India (ρ = 0.18, *P* = 1.1 × 10^−12^) and Bangladesh (ρ = 0.27, *P* = 3.1 × 10^−3^) (Supplemental Fig. S10). An explanation of the correlation based solely on multiple independent domestications would further require multiple such domestication events within each country, with local variability and structure preserved since the time of domestication—a very unlikely scenario. A more tenable hypothesis is substantial local gene flow between domesticated and wild rice in these regions.

**Figure 2.**
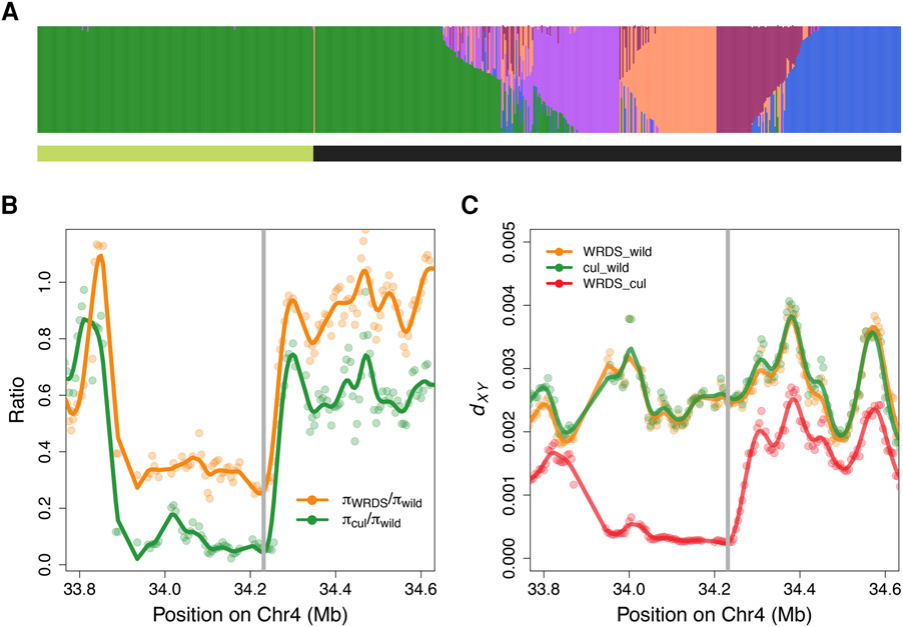
*sh4* haplotypes in wild and domesticated rice populations. (*A*) Local ancestry inference at *sh4* locus for *K* = 5. The bar at the *bottom* denotes *O. sativa* (green) and *O. rufipogon* (black) accessions, respectively. (*B*) Diversity reduction at the selective sweep region of *sh4*. The *y*-axis shows the ratio of pairwise differences estimator (π) of nucleotide diversity among populations. (*C*) *d*_XY_ values between populations at the selective sweep region of *sh4*. The gray line indicates the *sh4* gene region in *B* and *C*.

To further examine the hypothesis of gene flow between domesticated and wild rice populations, we analyzed the local ancestry at two known domestication-related genes, *sh4* and *PROG1*, and asked whether the domesticated alleles are found in the wild population or vice versa. These two genes were previously shown to be responsible for key morphological transitions from wild to domesticated rice: a mutation (G → T) in the coding sequence of *sh4* causes reduced shattering of rice grains (Li et al. 2006; Lin et al. 2007), and genetic variants in *PROG1* contribute to the transition from prostrate to erect growth in domesticated rice (Jin et al. 2008; Tan et al. 2008). To our knowledge, these are the only two genes in the rice genome that control critical traits distinguishing wild and domesticated rice; meanwhile, all domesticated rice share identical domesticated alleles at these loci (Lin et al. 2007; Tan et al. 2008), despite enormous allelic diversity commonly observed at other genomic loci among subgroups of domesticated rice. The domestication alleles confer traits strongly preferred by humans, but they are presumably highly deleterious in the wild: The nonshattering phenotype will increase the probability of herbivory of rice seeds, and erect growth will make rice plant more easily spotted and grazed by herbivores (Tan et al. 2008). We first examined the haplotype content at the *sh4* locus using a clustering approach (Methods; Supplemental Fig. S11). Despite varying *K* from 2 to 5, all domesticated rice accessions except the “misidentified” GSOR311586 remain assigned to a single component (Fig. 2A), suggesting they harbor closely related haplotypes, as previously argued (Tan et al. 2008). Surprisingly, 94 samples (21.6% of all wild samples) from the wild rice population are also consistently assigned to the domesticated cluster, suggesting that they have the domesticated allele at *sh4*. Using a PCR assay, we confirmed that all the assayed samples contained the derived allele (T) at the functional SNP position, supporting the local ancestry assignment method as an effective approach in discerning alleles (Supplemental Text S6; Supplemental Table S1). Since we adopted the 95% ancestry cutoff for identifying domesticated allele (Methods), the result suggests that 94 may represent a conservative estimate of the number of wild samples harboring the domesticated allele at *sh4*. This estimate is consistent with a previous study which determined that ∼27% of wild rice contain the nonshattering allele at *sh4* (Zhu et al. 2012).

The observation that these “wild” accessions contain the domestication allele at this key domestication gene can be explained by two hypotheses: introgression from domesticated rice or shared ancestral variation. In the first scenario, we would expect that these individuals might share the signal of the domestication-related selective sweep at the *sh4* locus and show a reduction in genetic distance to domesticated rice relative to the distance between other wild rice and domesticated rice at this locus. However, if these varieties harbor the domestication allele simply due to shared ancestry from pre-domestication, they should not show the signal of a domestication-related selective sweep. To test this hypothesis, we examined local diversity at this locus on wild rice carrying the domesticated allele of *sh4* (hereafter, WRDS) and found a fourfold reduction in relative nucleotide diversity across the 200-kb region that perfectly coincides with a similar diversity reduction in domesticated rice (Fig. 2B; Supplemental Fig. S12). Also, Tajima's *D* (Tajima 1989) is −2.63 in this region (Supplemental Fig. S15), indicating an excess of rare alleles relative to equilibrium expectations, which is also consistent with the scenario of a recent selective sweep. At the sweep region, the genetic divergence between WRDS and domesticated rice drops to 0 (Fig. 2C), indicating they share nearly identical haplotypes. However, the divergence between WRDS-wild and cultivated-wild population is consistently high and resemble background genomic levels (Fig. 2C; Supplemental Fig. S13). Taken together, these results show that the genetic similarity between WRDS and domesticated rice at *sh4* is caused by sharing of the same domestication allele transferred by gene flow from domesticated into wild rice populations. The fact that nominal wild rice has the shattering phenotype (Zhu et al. 2012), even when carrying the domesticated *sh4* haplotype, suggests that one or more compensatory mechanisms have evolved in wild rice populations in order to compensate for the extremely high influx of the domesticated *sh4* allele through continuous gene flow from domesticated rice.

When applying the same analysis to the *PROG1* locus, we identified 113 wild rice accessions (26.0% of all wild rice samples) carrying the domestication allele *prog1* (Methods; Supplemental Fig. S14); in these, the nucleotide diversity is reduced and Tajima's *D* is −2.42 (Supplemental Figs. S15–S18), similar to the pattern observed for *sh4*. A significant excess of these accessions (*n* = 66; *P* < 0.01, χ^2^ test) also carry the domestication allele at *sh4*. In total, 23 of 25 accessions in subgroup *Or-E* carry *prog1*, and 20 accessions carry the domesticated *sh4* allele. In the *Or-F* subgroup, 11 of the 12 accessions carry the *prog1* allele, and all of them harbor the domestication allele of *sh4*. When combined with the genome-wide admixture inferences, these results strongly argue that the *Or-E* and *Or-F* subgroups either emerged as a result of feralization of domesticated rice or have received very high levels of gene flow, most likely from the *aus* and *indica* varieties, respectively. Therefore, the shared ancestry of *Or-E*/*Or-F* with domesticated subgroups observed under the *K* = 9 should be interpreted as a consequence of extensive gene flow from domesticated rice. Moreover, it is noteworthy that 104 accessions of other subgroups of wild rice harbor the domesticated allele at either *PROG1* or *sh4*, resulting in a total 32% of annotated wild rice accessions carrying domestication alleles, suggesting that gene flow/feralization is substantial and not limited to only a subset of the wild rice subgroups (Supplemental Fig. S19).

Morphologically, domesticated rice has closed floret, making cross pollination difficult and keeping them largely self-fertilized. Wild rice, however, typically has open floret with exerted stigma, resulting in a higher rate of outcrossing, and this is mirrored by lower inbreeding coefficient estimates when compared with domesticated rice (*t*-test, *P* ≪ 0*.*01) (Supplemental Fig. S20). Thus, morphological differences predict an asymmetric pattern of gene flow, with its dominant direction from domesticated into wild populations. Moreover, the census sizes of domesticated rice populations are much larger relative to wild rice populations, which also suggests that gene flow will predominantly be from domesticated to wild rice. Consistent with these expectations, we find 207 domestication alleles at *sh4*/*PROG1* in wild rice populations, whereas the wild alleles in domesticated accessions are rarely observed (*n* = 3). Genome-wide admixture analyses are also consistent with this hypothesis: varying *K* from 2 to 9, we consistently observe domestication components in wild rice populations, but very little wild ancestry in domesticated rice (Suplpemental Fig. S1). For the *K* = 9 model, 50% of wild rice have >10% domesticated ancestry (Supplemental Fig. S21). Interestingly, wild rice populations are enriched with accessions containing 50%–60% or 90%–100% domesticated ancestry (Supplemental Fig. S21), possibly due to very recent gene flow.

### Geographic pattern of gene flow

**Figure 3.**
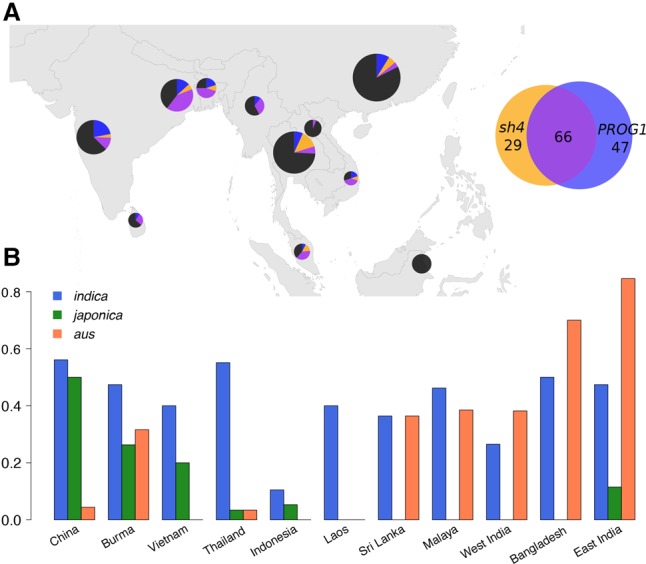
Geographic and subspecific pattern of gene flow. (*A*) Geographic distribution of domesticated alleles introgression at *PROG1* and *sh4* loci. Each pie chart represents the wild rice population of a region, and the area is proportional to its sample size. Each chart was divided into four categories according to the haplotype information at the two domestication loci: the domestication *sh4* allele only (yellow), the wild *prog1* allele only (blue), domestication alleles for both *sh4* and *PROG1* (purple), and wild *sh4* and *PROG1* alleles (black). Regions with less than 10 samples are not shown. (*B*) Geographic distribution of gene flow from *indica*, *japonica*, and *aus*. The proportions of admixed wild accessions with >5% ancestry of a certain subspecies in different regions are plotted. Wild accessions with *indica* have a pandemic distribution across rice cultivation regions, whereas accessions with *japonica* ancestry are endemic to regions including China, Burma, and Vietnam. Accessions with *aus* ancestry are mainly found in the Ganges Basin region, Sri Lanka, and Malaysia.

Monitoring the geographic pattern of the gene flow is important and may help guide the protection of wild rice germplasm. Using introgression of *sh4* and *PROG1* as an indicator, we found a significantly biased geographic distribution and could reject the hypothesis of a uniform amount of gene flow in all regions (*P* < 0.01, χ^2^ test) (Fig. 3A; Supplemental Table S2). In Bangladesh, 75% of wild accessions have domesticated alleles at one of the loci, and 45% have domestication alleles at both loci; in East India, we find 60.4% have one domesticated allele, and 41.5% have both. These numbers are much higher than the average level of 32.4% and 15.2%. In contrast, the northeast ranges of wild rice habitat show little or no introgression at either locus, e.g., only 17.5% of wild rice in China and 6.7% in Laos harbored domesticated alleles. In Indonesia, none of the rice accessions show evidence of introgression. Estimates of domesticated ancestry proportions (*K* = 9) in the genome of wild rice show a pattern of gene flow similar to that inferred using the two domestication loci (Fig. 3B; Supplemental Fig. S22). Varieties from Bangladesh have the highest proportion of domesticated rice ancestry among wild rice populations, with an estimate of 60% (Supplemental Fig. S22). The high level of gene flow in this region is consistent with field observations arguing that wild rice collected in this region may be heavily admixed (Morishima 2002). The neighboring regions of East India and Malaysia also have high estimates of 50% and 43% domesticated ancestry, respectively. In contrast, wild accessions from regions such as China, Laos, and Indonesia are relatively unadmixed, with domesticated admixture proportions as low as 8%, 10%, and 15%, respectively (Supplemental Fig. S22).

When examining gene flow from the perspective of the donors, we find a great difference in contribution from different domesticated rice subgroups, with 50% *indica*, 46% *aus*, and only 4% *japonica*. There are several factors likely contributing to this pattern. First, *indica* and *aus* varieties more readily shatter than *japonica* varieties (Konishi et al. 2006; Vaughan et al. 2008), so they are more likely to contribute to feralization. Second, wild rice is more likely to be sampled from areas in which varieties from the *indica* and *aus* subgroups are cultivated. *Japonica* varieties are mainly cultivated in the north, including North China, Korea, and Japan (G Khush, pers. comm.), where wild rice is rare and hence has not been included in wild rice sampling efforts. The extensive overlap of *indica/aus* planting area with wild rice habitat provides more opportunity for gene flow. In countries such as Laos, Vietnam, and Thailand, where gene flow mainly comes from the *indica* subgroup (100%, 94%, and 93%, respectively). However, in Bangladesh and India, gene flow is mostly contributed by the *aus* subgroup (75% and 55%, respectively). Interestingly, consistent with the broad distribution of *indica* cultivation, gene flow from the *indica* subgroup is present in wild populations from most geographic regions with an average of 50% of admixed samples carrying >5% *indica* ancestry (Fig. 3B). In contrast, *aus* and *japonica* are planted in more restricted geographic regions, and the distribution of gene flow into wild populations reflects these geographic biases (Fig. 3B). The proportion of wild accessions with >5% *aus* ancestry is high in Bangladesh and India (86% and 61%, respectively), which coincides well with the traditional planting area of *aus* varieties (Glaszmann 1987; Khush 1997). A considerable proportion of wild accessions from Malaysia and Sri Lanka (38% and 36%, respectively) also carry substantial *aus* ancestry. Wild accessions with >5% *japonica* ancestry are found in high proportions specifically in regions such as China, Burma, and Vietnam, representing the northeast range of wild populations where the planting region of *japonica* varieties and wild rice populations overlap.

**Figure 4.**
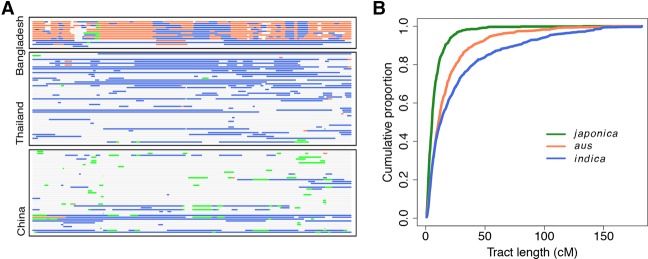
Distribution of chromosomal segments with domesticated ancestry in wild rice. (*A*) Ancestry assignment for wild rice from different regions. Each row represents a chromosome of one individual. Data for Chromosome 7 is presented. Bar colors indicate ancestry as follows: (gray) wild rice; (coral) *aus*; (blue) *indica*; (green) *japonica*. Introgression in Bangladesh is dominated by *aus*; Thailand is dominated by *indica*. Chinese wild rice harbors tracts of both *indica* and *japonica* ancestry. (*B*) Cumulative distribution of ancestry tract lengths from different subgroups of domesticated rice.

To determine whether gene flow from each domesticated subgroup occurred during the same or different time periods, we used local ancestry inference in admixed wild rice to identify introgressed domesticated chromosomal segments. Since the introgressed segments are broken into smaller segments by recombination over time, the distribution of introgressed tract lengths is informative about the age of admixture (Pool and Nielsen 2009; Moreno Estrada et al. 2013). The results of the local ancestry inference are consistent with our global ancestry inferences (Supplemental Fig. S23) and further support geographic biases in domesticated sources of gene flow (Fig. 4A). We summarized the length distribution of introgressed tracts from each domesticated subgroup and found that the length distribution of *japonica* haplotypes is enriched for smaller segments with an average of 8 centimorgan (cM) (Fig. 4B). The distribution of *japonica* haplotypes is significantly shorter than that of both *indica* (*t*-test, *P* < 1 × 10^−8^) and *aus* (*t*-test, *P* < 1 × 10^−8^), which have average haplotype length of 27 cM and 18 cM, respectively. This result indicates that the gene flow from *japonica* to wild rice is older than that of *aus* and *indica*.

### Feralization plays an important role in gene flow

**Figure 5.**
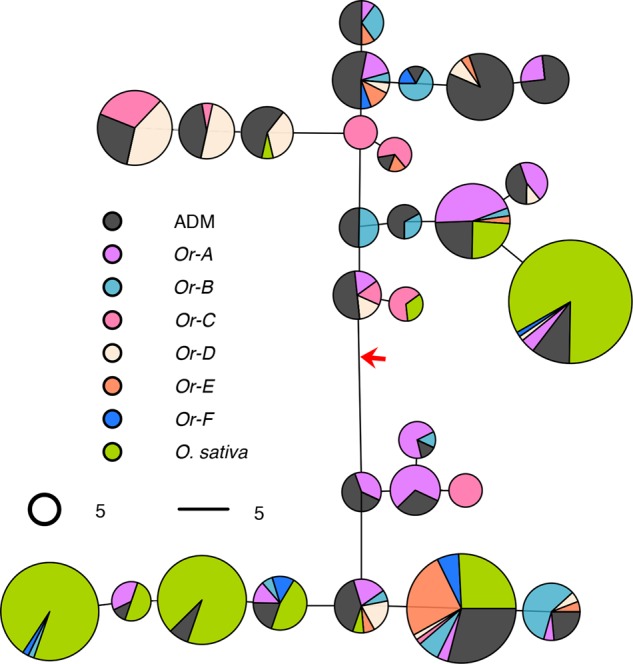
Chloroplast haplotype network among the 28 common haplotypes in rice primary gene pool. The haplotypes were defined using 74 common SNPs from the rice chloroplast genome. Each pie chart represents one haplotype, and it was further divided according to the subgroup information of the samples. All domesticated rice samples were colored in green, and wild rice samples were divided into seven subgroups. The root of the haplotype network was inferred using the chloroplast genome of *O. meridionalis* and is indicated by a red arrow. The length of lines connecting pie charts is proportional to the pairwise distance between haplotypes. The areas of the pie charts are proportional to the number of samples with the haplotype.

The gene flow from *O. sativa* to *O. rufipogon* may follow two different evolutionary pathways: pollen dissemination or seed dispersal. If seed spillage were involved, we would expect to find cytoplasmic genomes with domesticated rice haplotype in wild populations. In this study, we took advantage of the high copy number of the chloroplast genome, providing an average of 200× sequencing coverage for each accession in the sequencing data (Methods), to obtain highly accurate haplotype information. We first estimated a maximum likelihood (ML) phylogenetic tree of the chloroplast haplotypes (Supplemental Fig. S24). The domesticated rice samples were found in two clusters, corresponding to the *indica* and *japonica* subgroups. Interestingly, many wild rice chloroplast genomes were nested within the domesticated rice clusters. To further quantify the number of wild rice accessions that are closely related to domesticated rice chloroplast haplotype, we constructed a haplotype network using common polymorphic sites across rice chloroplast genomes (Supplemental Text S4), which summarizes all major chloroplast haplotypes in the primary gene pool of rice and the phylogeny among them (Fig. 5). Surprisingly, we found 98 accessions (28.8% of 340) of wild rice with identical chloroplast haplotypes to those of domesticated rice. For both *Or-E* and *Or-F*, which we have shown to carry domesticated nuclear ancestry, an excess of accessions harbor domesticated chloroplast haplotypes as well (17 of 24 for *Or-E*, *P* = 0.01; 8 of 12 for *Or-F*, *P* = 0.06, χ^2^ test). This further supports that these accessions in fact are established by seed dispersal, i.e., feral rice. These results suggest an evolutionary scenario that includes ancient feralization events followed by subsequent backcrossing with wild rice populations. In line with the analysis at domestication loci, gene flow from domesticated rice is not limited to just *Or-E* and *Or-F* subgroups, because domesticated chloroplast genomes are carried by other groups of wild rice as well (Supplemental Fig. S25).

### Selection and adaptation in feral rice

The exceptional relatedness of both nuclear and chloroplast genomes between *Or-E* and *aus* indicates that *Or-E* might have arisen from *aus* varieties in the very recent past and then diverged during adaptation to the local wild environments. Thus, a comparison of *Or-E* and *aus* genomes provides a unique opportunity to investigate the genetic basis of plant feralization. In order to identify loci that might have been differentially selected between domesticated and feral rice, we first scanned the genome using *F*_ST_ to identify highly differentiated genes between *Or-E* and *aus*. We performed gene ontology (GO) enrichment analysis on genes with *F*_ST_ values ranking in the top 5% of the empirical distribution. The top enriched GO terms are mostly high-hierarchy terms that are too general to provide any specific biological hints (Supplemental Table S3). However, among the top enriched GO terms that refer to explicit biological functions, abiotic and biotic resistance terms, including response to fungus (*P* = 8.1 × 10^−7^), bacterium (*P* = 1.5 × 10^−9^), salt (*P* = 8.4 × 10^−11^), cold (*P* = 4.8 × 10^−6^), and wounding (*P* = 3.6 × 10^−8^), are prominently enriched. This suggests that rice might have faced different biotic and abiotic selection pressures under domestic and wild conditions. Interestingly, the GO term “long-day photoperiodism” is also enriched, an enrichment which persists even if the GO analysis is limited to genes with the top 1% *F*_ST_ values, indicating that genes underlying flowering time in long-day condition are among the most differentiated genes between *Or-E* and *aus*. We subsequently identified genes under selection in *Or-E* that may have been targeted by natural selection during the feralization process. Interestingly, *HD1,* a gene underlying major quantitative trait locus (QTL) for photoperiod-dependent flowering (Yano et al. 2000), is among those with the most dramatic diversity reduction across *Or-E* rice genomes, ranking in the top 0.3% of diversity-reduction genes across *Or-E* rice genomes, suggesting strong selection on this locus in the *Or-E* population. A comparison of the haplotypes of *Or-E* and *aus* at this gene identified the most differentiated SNP as a nonsynonymous polymorphism (G/A, G387S) that is fixed for G in *Or-E* but has low allele frequency in *aus* (13.3%), a potential candidate causal mutation. It is likely that *HD1* is a target of selection for rice feralization and that the nonsynonymous mutation has contributed to the flowering time adaptation of rice in the wild habitat.

### Implications for rice domestication

The high level of gene flow between wild and domesticated rice has consequences for our understanding of the process of rice domestication. To illustrate this, we estimated admixture graphs of geographically defined wild rice and major groups of domesticated rice using TreeMix (Pickrell and Pritchard 2012), which uses a maximum likelihood (ML) method based on a Gaussian model of allele frequency change. We divided wild rice into five regional populations based on geographic characteristics of the wild rice area and potential boundaries between subgroups (Methods; Fig. 1B). Four major subgroups of domesticated rice were also included. Although the topology of the ML trees changes depending on the number of migration events (*m*) allowed in the model (Supplemental Fig. S26), certain patterns persist and are robust toward assumptions regarding *m*. First, the domesticated rice subgroups consistently show evidence of more genetic drift, likely because they underwent strong bottlenecks caused by the domestication process and by artificial selection. The *japonica* subgroups have exceptionally long branches consistent with the previously reported much stronger bottleneck in their domestication history (Caicedo et al. 2007; Zhu et al. 2007; Gao and Innan 2008). Wild rice populations in the Ganges Basin (GBW) consistently form a clade with *indica* and *aus* (Supplemental Fig. S26). Two hypotheses could explain this pattern: (1) *indica* and *aus* were domesticated from the GBW very recently, or (2) as suggested by the previous analyses in this manuscript, the GBW populations are a product of feralization from domesticated *aus* and *indica* rice. Similarly, *temperate* and *tropical japonica* forms a clade with Chinese rice when assuming no migration.

**Figure 6.**
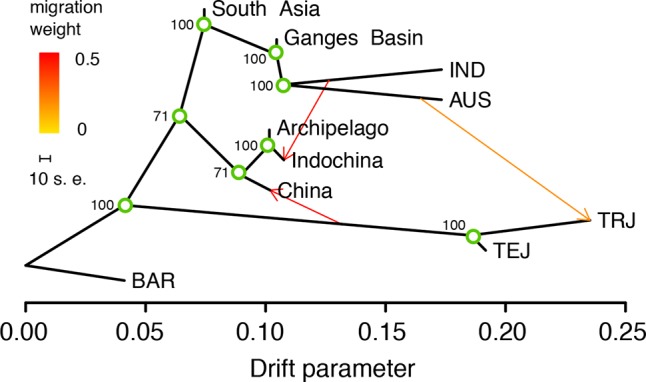
Maximum-likelihood admixture graph on the primary gene pool of Asian domesticated rice. The wild rice (*O. rufipogon*) population was divided into five geographic populations (Methods). The abbreviations for the major domesticated rice subgroups are the same as in Figure 1. African wild rice, *O. barthii* (BAR), was used to root the tree. The bootstrap values on the tree are based on 1000 replicates. Arrows on the graph represent admixture events among different rice populations.

Allowing just one migration event (*m* = 1) (Supplemental Fig. S26), we observe an admixture from *indica* into the Indochina wild rice population (ICW) contributing 46% of the DNA in Indochina. This is consistent with the results that a substantial amount of *indica* ancestry is observed in ICW (Figs. 1B, 4A). Allowing two admixture events (*m* = 2) (Supplemental Fig. S26), a substantial amount of gene flow from Indochina to China is observed. This is possibly a consequence of Chinese wild rice being admixed between original wild rice and domesticated rice. This is supported by the fact that when *m* = 3, wild Chinese rice groups cluster with wild rice in Indochina and the Archipelago, but with substantial gene flow (49%) from the ancestor of *japonica* (Fig. 6). Likely, the true wild ancestor of *japonica* rice is not represented in the sample by any current wild descendant population. The *Or-B* component found in China may not be an “authentic” wild component, but rather it is a product of admixture between wild rice and ancient *japonica*. The wild rice ancestral to the domesticated *japonica* may be, in fact, already extinct. For models with *m* = 3, we observed an admixture event, with a proportion of 19%, from *aus* to *tropical japonica* (Fig. 6), indicating substantial genetic ancestry shared between these two subgroups. We consistently observe *japonica* sharing high residuals with *aus/indica* (Supplemental Fig. S26), which likely reflects that they share many genomic components caused by hybridization in their domestication and breeding history.

## Discussion

Elucidating the pattern of gene flow among wild and domesticated rice is important for understanding the history of rice domestication. Multiple studies argued for the independent domestication of rice based on reciprocal monophyly of *indica* and *japonica* when using different nuclear DNA markers in independent rice collections (Cheng et al. 2003; Zhu and Ge 2005; Rakshit et al. 2007; Civáň et al. 2015). In contrast, treating *O. rufipogon* as a single homogenous group in an analysis of divergence times and population trees, Molina et al. (2011) argued for a single domestication event. Based on comparisons to wild rice samples, Huang et al. (2012b) similarly argued that rice domestication originated in South China. Recently, Civáň et al. (2015) argued that the *aus* group had been independently domesticated in the Ganges Basin area. In this study, we showed that there is extensive, continuous gene flow from domesticated rice into wild rice populations. It suggests that the patterns described in previous studies are likely caused by gene flow from domesticated rice into wild rice populations after rice domestication. We show that wild rice in the Ganges Basin is likely feral rice, recently diverged from domesticated rice, and Chinese wild rice has received extensive gene flow from an ancient *japonica* population. Furthermore, the *indica* and *aus* groups are always sister groups, suggesting a single domestication event for these two groups. TreeMix results are largely compatible with a dual origin of domestication given the deep divergence observed between *indica* and *japonica* subgroups. The divergence even spans the diversity of present-day Asian wild rice, but we caution that current wild rice samples may be biased due to incomplete sampling or loss of “authentic” wild rice samples in germplasm centers during preservation. We cannot exclude the possibility of a single domestication hypothesis because the deep divergence could also be caused by substantial independent gene flow from other wild rice species into different domesticated rice subgroups, which is practiced in rice breeding (Brar and Khush 1997). The single domestication hypothesis would require either (1) extensive gene flow from wild rice into the *indica/aus* subgroups so that their genomes now are dominated by gene flow from wild rice, combined with a subsequent loss (or lack of representation) of true “ancestral” wild rice; or (2) a single domestication hypothesis could also be compatible with the data if all wild rice populations represented in the panel are dominated by gene flow from local domesticated rice occurring continuously over the past ∼9,000 yr. However, the dual domestication model is arguably a simpler scenario.

Rice was introduced into the United States <400 yr ago, and rice cultivation was not widely expanded until the 1750s (Dethloff 2003). However, weedy rice is now common in rice growing regions in the United States and is one of the major weeds limiting rice production (Ziska et al. 2015). Genetic analysis has shown that American weedy rice population arose independently from *indica* and *aus* varieties (Londo and Schaal 2007). These observations indicate that rice could frequently revert to the wild state in domestication traits. Weedy rice is a conspecific form of cultivated rice, while displaying distinguishing features including shattering grains and strong seed dormancy typical of wild rice (Ferrero 2003; Song et al. 2014). The shattering and seed dormancy phenotypes acquired in weedy rice are presumably adaptive in wild conditions, potentially further facilitating feralization. A crop–weed–wild complex is found throughout regions where wild and cultivated rice overlap, and gene flow among components within the species complex is frequently observed (Ellstrand et al. 2013; Pusadee et al. 2013, 2016; Song et al. 2014). In Asia, rice cultivation has been performed for thousands of years, and rice feralization has likely happened throughout this period as well (Vaughan et al. 2005). In fact, much presumed wild rice in many parts of Asia may possibly be descendants of ancient feralization/hybridization events. Wild and weedy rice found all over the world might simply represent different stages of the feralization process. It is even possible that what we today characterize as wild rice in Asia, may largely be feral rice that has undergone thousands of generations of natural selection in the wild, and the original species from which *O. sativa* was domesticated is either extinct or has been almost entirely overwhelmed by the massive amounts of gene flow from domesticated rice. *O. rufipogon* may then represent a nominal species created by human domestication and subsequent feralization/hybridization.

## Methods

### Genomic data acquisition

The genomic data of wild rice was downloaded from the European Nucleotide Archive under the accession number ERP001143 (Supplemental Text S2). Domesticated rice data was downloaded from the NCBI BioProject Repository (project number: PRJNA301661).

### Short-read mapping

Reads were mapped to the rice genome (IRGSP-1.0) (Kawahara et al. 2013) with BWA (version 0.7.0) (Li and Durbin 2009), and the mapping was further improved with Stampy (version 1.0.20) (Lunter and Goodson 2011). PCR duplicates were removed by “rmdup” in SAMtools (version 0.17) (Li et al. 2009). We realigned reads at gapped regions with GATK (version 2.6) (DePristo et al. 2011).

### Population structure, phylogeny, network, and TreeMix analyses

We estimated genotype likelihoods of populations with the “-GL” option in ANGSD (version 0.542) (Korneliussen et al. 2014). Inbreeding coefficients for each individual were calculated using a probabilistic framework implemented in ngsF (Vieira et al. 2013). The variability and allele frequency of each genomic site was estimated by ANGSD using the “-doMaf” command. Variable sites were extracted and used for further analyses. A genotype likelihoods-based method, implemented in NGSadmix (Skotte et al. 2013), was used for global ancestry inference. The analysis was conducted on the combined population, including 203 domesticated and 435 wild rice accessions. We randomly picked one variable site for every 5-kb genomic region from variable sites to reduce effects of linkage disequilibrium. In total, 60,722 evenly distributed markers were used. With these markers, we successively tested 14 clustering models in the population with *K* (presumed cluster number) ranging from 2 to 15. For each *K*, we ran 200 independent replicate optimizations, picked the clustering model with the highest log likelihood value, and the corresponding log likelihoods are shown in Supplemental Figure S3. PCA was performed with the same genotype likelihoods data set using ngsCovar from the ngsTools package (Fumagalli et al. 2014). All plots were generated with R (version 3.0.2) (R Core Team 2016). We estimated admixture trees, phylogenies, and haplotype networks using standard methods explained in Supplemental Text S4 and S5.

### Introgression analyses at two domestication loci

To identify domestication haplotypes at the *sh4* locus in wild rice, we inferred local ancestry in a 10-kb region centered on *sh4*. Using genotype likelihoods, we ran NGSadmix for varying values of *K,* and domesticated rice accessions were consistently assigned to one component from *K* = 2 to *K* = 5 except for the misidentified sample, GSOR311586. At *K* = 6, the domesticated rice population splits into two major components, which conflicted with prior knowledge that there is one haplotype at this locus in the domesticated rice population (Li et al. 2006; Lin et al. 2007), suggesting that *K* = 6 model is overfitting. To further investigate this issue, we randomly sampled three samples from each domesticated rice population assigned to different ancestries under the *K* = 6 model and PCR amplified the *sh4* locus in these accessions. They turned out in all cases to harbor the domesticated allele at the causal variant site. Consequently, we proceeded to use the *K* = 5 model for allele identification of the domestication haplotype. Wild rice samples with at least 95% domesticated ancestry at the locus were inferred to carry the domesticated allele. For the *PROG1* locus, there is also a strong selective sweep (He et al. 2011), and all domesticated rice share identical haplotypes in this region (Tan et al. 2008). We thus applied the same procedures to identify introgression at this locus. For both genes, we confirmed that the domesticated haplotypes identified from the wild rice population contained the domesticated allele at the functional SNP site through PCR amplification (Supplemental Text S6; Supplemental Table S1).

Tajima's *D* and *θ*_*π*_ statistics were calculated under a probabilistic framework designed for low-coverage data (Korneliussen et al. 2013). The methods are implemented in ANGSD and can be invoked by parameter “-doThetas.” *d*_XY_ between populations was calculated in 10-kb nonoverlapping windows. For each window, *d*_XY_ values were calculated for all paired polymorphic sites and then averaged over sites. For each polymorphic site, the allele frequencies in population X and Y are denoted as *p*_X_ and *q*_X_, and *p*_Y_ and *q*_Y_, respectively, and *d*_XY_ for a site is calculated as *d*_XY_ = *p*_X_*q*_Y_ + *p*_Y_*q*_X_.

### Genotype calling and local ancestry inference

To infer the local ancestry in admixed wild rice genomes, we first set up reference wild, *temperate japonica*, *aus* and *indica* panels. Under the *K* = 9 admixture model, wild rice individuals whose ancestry were inferred to be ≥80% from one of four wild rice specific components, and which contained neither *prog1* nor domesticated allele of *sh4,* were used in the reference wild rice panel. Domesticated rice with ≥80% inferred ancestry from one of *indica*, *aus*, or *temperate japonica* was used as reference *indica*, *aus*, or *temperate japonica* panel, respectively. Wild rice samples with combined ancestry of *indica*, *aus*, and *temperate japonica* ≥20% were included as admixed accessions. Local ancestry assignment was performed on admixed rice genomes with RFMix (Maples et al. 2013). Since this algorithm uses haplotypes as input, we called genotypes in both admixed and reference panel samples with ANGSD (Korneliussen et al. 2014). Imputation and phasing was further performed on the data sets with BEAGLE (version 3.3.2) (Browning and Browning 2007).

### Selection detection in feral rice

We calculated *F*_ST_ between the *Or-E* and *aus* populations for all rice genes using ngsTools (Fumagalli et al. 2014), which calculates *F*_ST_ using genotype likelihoods, taking genotyping uncertainty into account. We performed GO analysis on the ranked gene list based on the *F*_ST_ values: GO annotation of all rice genes was downloaded from Gramene (http://www.gramene.org/; release 49), the enrichment of each GO was tested using Fisher's exact test, corrected for multiple tests using a Bonferroni correction. The significantly enriched GO terms for the top 5% *F*_ST_ genes can be found on Supplemental Table S3. Nucleotide diversity reduction in both *aus* and *Or-E* genomes was estimated by comparing with diversity in wild rice populations. The diversity for each population was estimated using the “-doThetas” command in ANGSD (Korneliussen et al. 2014).

## Data access

PCR-amplified sequences have been submitted to NCBI GenBank (https://www.ncbi.nlm.nih.gov/genbank/) under accession numbers KY701787-KY701861 (for *sh4*) and KY701862-KY701970 (for *PROG1*).

## Supplementary Material

Supplemental Material
