# Supplementary material for: Asian wild rice is a hybrid swarm with extensive gene flow and feralization from domesticated rice
Source: Genome Res. 2017 Jun;27(6):1029–38. doi: 10.1101/gr.204800.116 (PMC5453317; doi:10.1101/gr.204800.116)
Supplement: Supplemental Material [file supp_27_6_1029__index.html]

Asian wild rice is a hybrid swarm with extensive gene flow and feralization from domesticated rice — Supplemental Material 

# Asian wild rice is a hybrid swarm with extensive gene flow and feralization from domesticated rice

## Supplemental Material

- Supplemental\_Table\_S1.xls
- Supplemental\_Table\_S2.xls
- Supplemental\_Table\_S3.xls
- Supplemental\_Text\_Figures.docx
