# Supplementary material for: Asian wild rice is a hybrid swarm with extensive gene flow and feralization from domesticated rice
Source: Genome Res. 2017 Jun;27(6):1029–38. doi: 10.1101/gr.204800.116 (PMC5453317; doi:10.1101/gr.204800.116)
Supplement: Supplemental Material [file supp_gr.204800.116_Supplemental_Text_Figures.docx]

# Supplementary Notes

## Supplementary Text S1.

**Wild rice material**

The 446 *O. rufipogon* samples were collected during 1957 to 1997 in three research surveys. The first research survey was led by Dr. H. I. Oka (http://shigen.nig.ac.jp/rice/oryzabase/ricereport/), and most of the studies involved in the survey were summarized in the book “Origin of Cultivated Rice” ([Oka 1988](#_ENREF_11)). To expand wild rice collection in different habitats, the second survey led by Dr. H. Morishima was focused on wild rice collection in Southeastern Asia (http://shigen.nig.ac.jp/rice/oryzabase/ricereport/). The third survey led by Dr. Y. I. Sato was focused on wild rice collection in Indo-China area that has been left behind in previous two surveys (http://shigen.nig.ac.jp/rice/oryzabase/ricereport/). The sampling location of this wild rice collection fully reflects the habitat for extant wild rice, representing an invaluable resource for studying genetic diversity of wild rice.

The wild rice samples have been preserved in the germplasm center of National Institute of Genetics since first being collected. Among all the samples, ~20% of them do not bear enough seeds and are thus preserved by roots (Zi-Xuan Wang, personal communication). The rest samples are propagated by seeds every 5-10 years. Each propagation was conducted in a enclosed greenhouse that is remote from cultivated rice to prevent possible hybridization between wild rice and cultivated rice. To further prevent potential hybridization between different wild rice samples, each wild rice strain was bagged on the top before panicle emergence to ensure inbreeding (see <http://shigen.nig.ac.jp/rice/oryzabase/strain/wildCore/protocol> for cultivation method for wild rice).

Even though substantial efforts have been devoted to comprehensive sampling and preservation of this wild rice collection, we caution that these wild rice may still be a biased representation of genetic diversity of all living wild rice due to: (1) these wild rice samples may represent an incomplete sampling of all living wild rice, and (2) genetic diversity may have lost during rice preservation.

## Supplementary Text S2.

**Genomic data of wild rice**

Among 446 wild rice (*O. rufipogon*) samples, 6 samples did not have available sequencing data, 5 samples had too little sequencing data (reads file <100Mb) or corrupted data, and were thus excluded from analyses. In total, genomic data of 435 wild rice samples was downloaded and included for downstream analysis (Supplemental Table S1).

## Supplementary Text S3.

**Fitting Admixture Models**

When assuming a two-cluster (*K* = 2) model, the population was split into *indica* and *japonica* components, reflecting the *indica-japonica* differentiation in the primary gene pool. Previous studies have identified five subgroups (*indica, aus, aromatic, tropical japonica* and *temperate japonica*) in domesticated rice by synthesizing information from the geographic distribution, phenotypes, and genetic markers ([Garris et al. 2005](#_ENREF_2); [Li et al. 2010](#_ENREF_10); [Zhao et al. 2010](#_ENREF_20); [Huang et al. 2012b](#_ENREF_4); [Xu et al. 2012](#_ENREF_18)). In our combined data from wild and domesticated rice, the value of *K* in which these five established groups are maintained, is *K* = 9 (Fig. [1](#_bookmark48)A; Supplemental Fig. S2-S3), and we base our discussion on this value of *K*, although none of our general conclusions are sensitive to the exact choice of *K*. We chose this criterion because it allows us to cluster wild rice accessions in a manner that is consistent with the accepted categorization of domesticated rice. At higher *K*, *aus* is divided into two components (Supplemental Fig. S4). Under the *K* = 9 model, 81% of domesticated rice individuals fall into one of the five domesticated rice subgroups and the rest are admixed using an 80% ancestry cutoff. The categorization is consistent with the assignment results based on domesticated rice samples only (Supplemental Fig. S5) but, as mentioned in the main text, still reveals substantial gene-flow from wild rice.

The *aus* subgroup is assigned to a single genetic component when the number of assumed ancestral populations (*K*) ranges from 4 to 9. When *K* is greater than 9, a new component is identified from the *aus* subgroup and, at the same time, the *Or-E* group is assigned to this new component. This reflects further genetic differentiation within *aus* subgroups then that between *aus* and *Or-E*. At *K* = 15 (Supplemental Fig. S2), a new subgroup, which is termed *aus-b* (the other subgroup in *aus* is termed *aus-a*), emerges from *aus* varieties. Interestingly, *aus-a* and *aus-b* have distinct geographic distributions (Supplemental Fig. S4): all *aus* varieties from Ganges Basin (including Bangladesh and East India) belong to *aus-a*, while all varieties in Sri Lanka (4/4) belong to *aus-b*.

## Supplementary Text S4.

**Chloroplast phylogeny and haplotype network**

The genomic sequencing data for each rice sample was mapped to the rice chloroplast genome using the procedures described above. Individuals with a genome wide coverage *≥*100X (approximately 70 times of the nuclear genome wide coverage) were retained for downstream analyses. In total, 391 wild rice and 193 domesticated rice samples were included. We called genotypes with “samtools mpileup” and parameters “-baq” and “-C50” to reduce false positive calls introduced by mis-mapping at gap regions. Genotypes with posterior probabilities greater than 0.95 were retained. Inverted Repeats (IR) and 100bp flanking regions of the chloroplast genome were masked since identical sequences in the reference cause ambiguous read mapping. Heterozygous sites were removed. We further control missing data rate by filtering all sites where missing data rate is greater than 50%. In total, we analyzed 87,482bp of aligned sequence data containing 480 polymorphic sites. The Maximum Likelihood Tree (MLT) based on the aligned sequences was obtained using RAxML (version 8.2.X) ([Stamatakis 2006](#_ENREF_14)). To reduce the computational burden, we adopted the GTRCAT model which is an empirical approximation for the widely used General Time Reversible model of nucleotide substitution under the gamma model of rate heterogeneity (GTRGAMMA). The ML bootstrap values were based on 200 replicates and tree visualization was performed with MEGA4 ([Tamura et al. 2007](#_ENREF_15)).

For network reconstructions, we excluded low-frequency SNPs (MAF <5%) and, after the filtering, 74 sites were kept. In this dataset, the missing data rate is 5%. We then imputed the missing data with Beagle ([Browning and Browning 2007](#_ENREF_1)). Based on the information at the 74 sites, the haplotypes in the samples were categorized into 56 groups, among which 28 groups consist of no more than 6 six individuals for each group and were thus excluded for network construction. We constructed the network with the rest 28 haplotypes which covers 90.8% (530/584) individuals from the combined population, using the R package pegas ([Paradis 2010](#_ENREF_12)). The root of the haplotype network was inferred with the haplotype of *O. meridionalis* sample. In the domesticated rice population, 10 haplotype groups were constructed, among which three groups consist of only one sample for each group and were thus excluded from the domesticated chloroplast haplotypes. Notably, two of the three excluded samples also contain wild allele at either *sh4* or *PROG1*.

## Supplementary Text S5.

**TreeMix analyses**

To further investigate possible gene-flow between different subgroups within the rice primary gene pool, TreeMix ([Pickrell and Pritchard 2012](#_ENREF_13)) was used to construct a population Maximum-likelihood (ML) tree and simultaneously estimating migration events. To account for the geographic structure of wild rice habitats and potential genetic boundaries between wild rice subgroups (Fig. 1B), we divided wild rice into five regional populations: South Asia population (SAW) includes samples from West India (West of 85E), Nepal and Sri Lanka; Ganges Basin population (GBW) includes East India, Bangladesh; Indochinese Peninsula population (ICW) include Burma, Cambodia, Laos, Thailand, Vietnam; Archipelago population (APW) include Indonesia, Philippines, Malaysia, Australia, Australian New Guinea, Dutch New Guinea; Chinese population (CNW) include all wild rice samples from China. Four major cultivated rice subgroups: *indica* (IND), *aus* (AUS), *tropical japonica* (TRJ) and *temperate japonica* (TEJ). To account for inherent uncertainty in low coverage data, we estimated allele frequencies for each subpopulation using the ANGSD “-doMafs” command ([Korneliussen et al. 2014](#_ENREF_7)). We only retained polymorphic sites for which 40% of the individuals in each subgroup have sequencing read support. After filtering, 2,580,532 SNP sites were used for the tree construction. For each TreeMix run, we concatenate 7,000 SNP sites as a block, which corresponds to a ~1Mb genomic region which exceeds the range of linkage disequilibrium in rice ([Zhao et al. 2011](#_ENREF_19)). We used *O. barthii* (African wild) to root the tree, and population genomic sequencing data were downloaded from NCBI Short Reads Archive (SRR1206363-SRR1206371) ([Wang et al. 2014](#_ENREF_17)). The bootstrap values on the tree are based on 1,000 replicates.

## Supplementary Text S6.

**PCR assays**

To confirm whether the domesticated alleles from wild rice identified using local ancestry assignments were real, we asked whether the identified wild rice samples contain a domesticated allele at the causal functional mutation sites. We thus PCR amplified short genomic sequences covering the causal mutation sites, and then mapped the amplified sequence to rice reference genome (IRGSP-1.0) ([Kawahara et al. 2013](#_ENREF_6)) using BWA ([Li and Durbin 2009](#_ENREF_9)). The genotyped sites were Chr4:34232985 (C/A) and Chr7:2839526 (T/A) for *sh4* ([Li et al. 2006](#_ENREF_8)) and *PROG1* ([Jin et al. 2008](#_ENREF_5)), respectively. Of the 74 samples genotyped for *sh4*, all of them were confirmed to contain domesticated allele; 106 out of 109 samples genotyped for *PROG1* harbor domesticated allele (Supplemental Table S1).

PCR primers used for amplification of *PROG1* are PF: 5’-CTGACAGTACGTTGCTGT-3’ and PR: 5’-CATGGTGATCGTGGAGAT-3’. The PCR thermal cycles are set as: 94*◦*C, 2 min; 35 X (98*◦*C, 15 sec; 58*◦*C, 30 sec; 68*◦*C, 1.5 min); 68*◦*C, 5 min. PCR primers for *sh4* are SF: 5’-GAGAAGAGAGCGAAGTAG-3’ and SR: 5’-ATCCGATGCCTCGATCCAT-3’. The PCR thermal cycles are: 94*◦*C, 2 min; 35×(98*◦*C, 15 sec; 58*◦*C, 30 sec; 68*◦*C, 1.5 min); 68*◦*C, 5 min. High fidelity DNA polymerase (KOD FX, Toyobo, Japan) was used for the PCR reactions. The sequence of each DNA sample were determined with single pass Sanger sequencing.

# Supplementary Figures

## Supplementary Figure S1.

Ancestry inference in the rice primary gene pool with *K* varying from 2 to 9. The estimation is based on genotype likelihoods at 60,722 polymorphic sites that are randomly selected from the rice genome and are spaced at least 5kb apart. The color bar at the bottom indicates different rice subgroups, and all wild rice are denoted by black bar.

## Supplementary Figure S2.

Admixture analysis in rice primary gene pool with *K* ranging from 10 to 15. The ordering of the individuals is the same as in Supplemental Fig. S1.


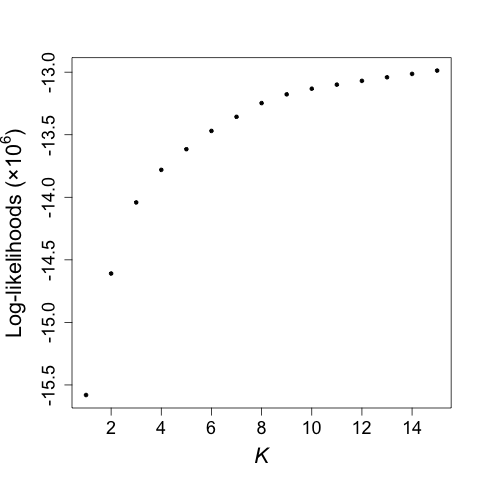


## Supplementary Figure S3.

Log-likelihoods for ancestry inference models under different presumed populations (*K*) in rice primary gene pool. As expected, likelihoods keeps increasing from *K* = 1 to *K* = 15 because it increases the model fitting by adding more parameters.

## Supplementary Figure S4.

Geographic distribution of *aus-a* and *aus-b*. *Aus* samples are represented in the map with pie charts. The area of the charts are proportional to the sample size. Samples out of Asia are summarized with the charts at the lower left of the map. The color codes are: orange for *aus-a* and blue for *aus-b*.

## Supplementary Figure S5.

The concordance of subgroup assignments between previous ([Wang et al. 2016](#_ENREF_16)) and this study. In previous assignment, the domesticated rice population was categorized into 6 groups. For individuals in each group, their assignments in this study were indicated by different colors. In subgroup *temperate japonica*, *tropical japonica* and *aromatic*, there are perfect concordance. In subgroup *indica* and *aus* of previous study, 1 and 6 individuals were assigned as *admixed* in this study, respectively, while one *admixed* individual (Supplemental Fig. S6) was assigned to *Or-C* in the current study.


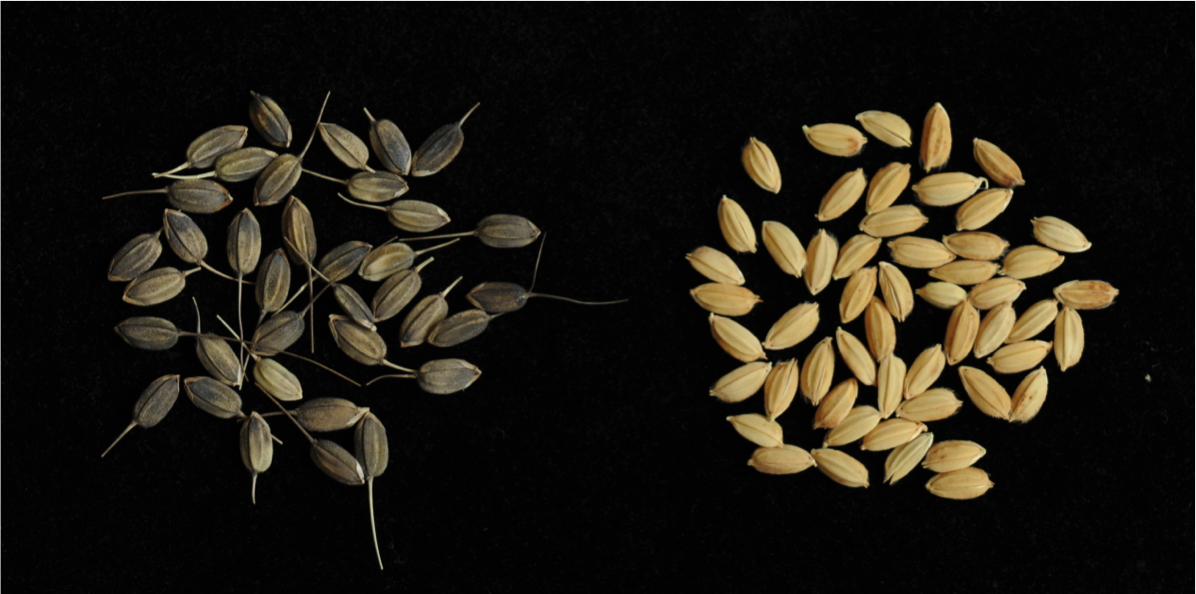


## Supplementary Figure S6.

The rice grains of GSOR311586 (left) and Nipponbare (right). The seeds of GSOR311586 have black hull with long awn at maturation.

## Supplementary Figure S7.

The assignment of subgroups for *O. rufipogon* in the current study and in the study by Huang *et. al.* ([Huang et al. 2012a](#_ENREF_3)). In Huang *et. al.*, wild rice was divided into three subgroups, denoted as Or-I, Or-II and Or-III, and Or-III was further divided into Or-IIIa and Or-IIIb. In the current study, wild rice populations were divided into 6 subgroups: *Or-A*, *Or-B*, *Or-C*, *Or-D*, *Or-E* and *Or-F*. Among these subgroups, *Or-A* corresponds to Or-II; *Or-B* corresponds to Or-IIIa; *Or-C* corresponds to Or-IIIb; *Or-D*, *Or-E* and *Or-F* correspond to Or-I. Individuals identified to be admixed are found in all of the three subgroups identified in Huang *et. al.*. The numbers on the graph represent numbers of accessions in each category.

## Supplementary Figure S8.

Principal component analysis in rice primary gene pool. The left panel shows the plot of PC1 and PC3, the right panel shows the plot of PC1 and PC4.


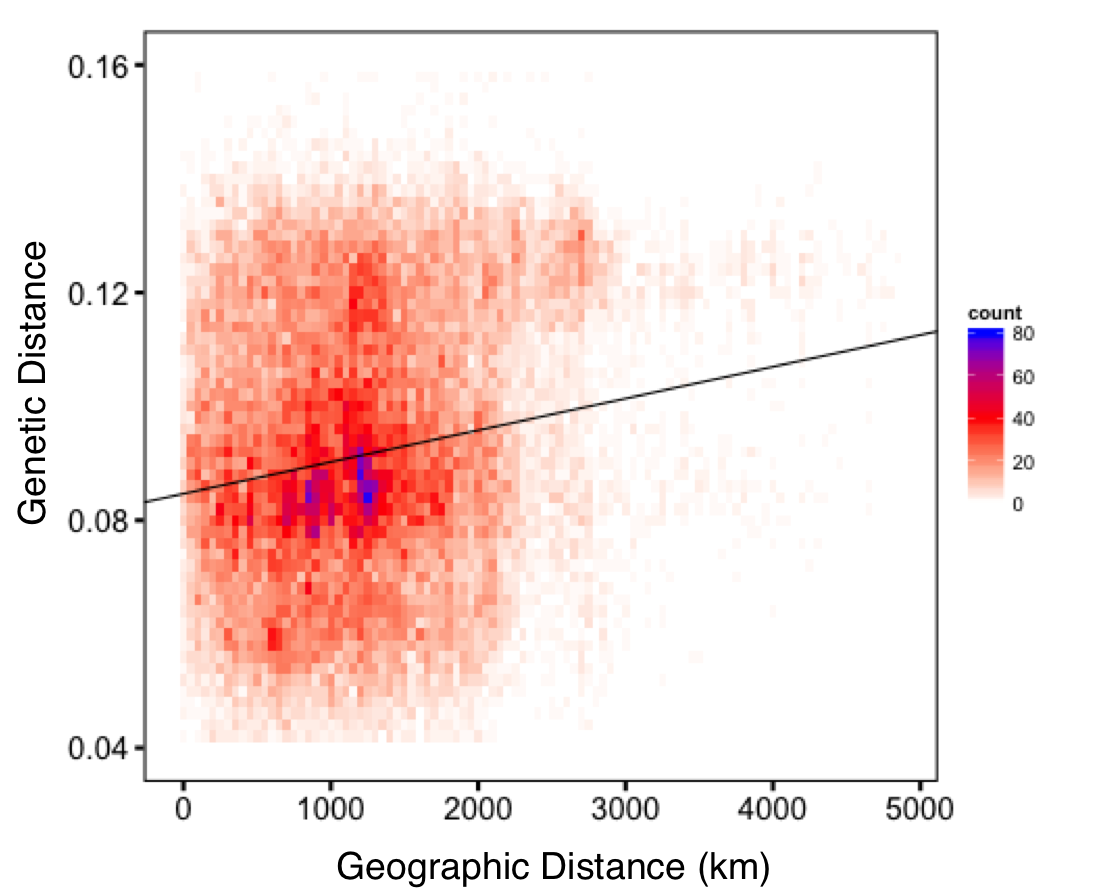


## Supplementary Figure S9.

Correlation between geographic and genetic distance in all studied wild and domesticated rice samples.

## Supplementary Figure S10.

Correlation between geographic and genetic distance in wild and domesticated rice population in (A) Bangladesh and (B) India.

## Supplementary Figure S11.

Local ancestry inference in the *sh4* region Ancestry inference was performed using genotype likelihoods from a 10kb region containing *sh4*.

## Supplementary Figure S12.

The relative nucleotide diversity (compared with wild rice population) in WRDS and cultivated population across the rice genome. The *sh4* gene region is indicated by a vertical line.

## Supplementary Figure S13.

*d*_XY_ values between populations across rice genome. The *sh4* gene region is indicated by a vertical line.

## Supplementary Figure S14.

Inferred ancestry proportions around the *PROG1* region. Ancestry inference was performed using genotype likelihoods from a 10Kb region containing *PROG1*.

## Supplementary Figure S15.

Tajima’s *D* statistics in the regions surrounding domestication-related loci. The left panel shows Tajima’s *D* value at the gene and flanking region of *sh4*, the right panel shows that of *PROG1*. The grey lines indicate the coding region of the two genes, respectively.

## Supplementary Figure S16.

Population genetic analyses of the *PROG1* region. (A) ancestry proportions around the *PROG1* region using the *K* = 5 model. We identified two domesticated rice accessions (GSOR311644 and GSOR311586; indicated by the red arrow) that harbor the wild allele at this locus, and this is confirmed by PCR amplification. (B) shows diversity reduction in wild rice with domesticated *PROG1* allele (WRDP) and cultivated rice population at the selective sweep region of *PROG1*. (C) shows *d*_XY_ values at the selective sweep region of *PROG1*. Grey line indicates the *PROG1* gene region.

## Supplementary Figure S17.

The relative nucleotide diversity (compared with wild rice population) in WRDP and cultivated population across the rice genome. The *PROG1* gene region is indicated by a vertical line.

## Supplementary Figure S18.

*d*_XY_ values between populations across rice genome. The *PROG1* gene region is indicated by a vertical line.

## Supplementary Figure S19.

Nuclear genomic ancestry of ‘wild’ rice samples containing the domesticated allele at either *PROG1* or *sh4*. The ancestry of *O. rufipogon* accessions was determined using *K* = 9 model (Supplemental Fig. S1).

## Supplementary Figure S20.

Inbreeding coefficient (*F*) estimates for *O. rufipogon* and *O. sativa*.


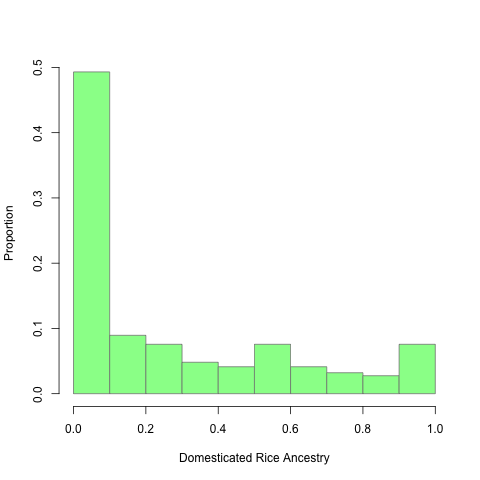


## Supplementary Figure S21.

Domesticated rice ancestry in wild rice. The histogram shows the proportion of wild rice accessions with varying proportion of domesticated rice ancestry.

## Supplementary Figure S22.

Geographic distribution of gene flow. The amount of gene flow was estimated from nuclear genomic ancestry. The ancestry in each location is first divided into domesticated (light green) and wild (black) ancestries. Then the domesticated ancestry is further divided into parts that are contributed by *indica* (blue), *aus* (coral) or *japonica* (dark green), respectively.

## Supplementary Figure S23.

Comparison of ancestry analysis with RFMix and NGSadmix in *O. rufipogon* samples which are inferred to have *>*20% domesticated rice ancestry. Ancestry inferences with NGSadmix under *K* = 9 model (A) and RFMix (B) are consistent. Each line in the plot represent the ancestry inference of one individual. Color code: blue for *indica*, green for *japonica*, coral for *aus*, and grey for wild rice.

## Supplementary Figure S24.

Maximum-likelihood (ML) phylogenetic analysis with chloroplast genomes of domesticated and wild rice. The ML tree was constructed with whole chloroplast genome marker. It was rooted with *O. meridionalis* chloroplast genome. Each color bar in the leaf of the tree corresponds to one sample. Samples with similar haplotypes are merged into triangles; and the color of triangle is decided by the composition of domesticated and wild rice in the group. Bootstrap values are indicated at the tree nodes.

## Supplementary Figure S25.

Nuclear genomic ancestry of ‘wild’ rice samples containing domesticated chloroplast. The haplotypes of the chloroplast were determined by genotypes and the nuclear ancestry was determined under *K* = 9 model (Supplemental Fig. S1).

## Supplementary Figure S26.

Maximum-likelihood tree and corresponding residual fit on the primary gene pool of Asian domesticated rice assuming 0 to 3 migration edges. African wild rice *O. barthii* (BAR) is used to root the tree. The wild rice population was divided into 5 geographic populations (see Methods), and the abbreviations for domesticated rice are as follows: IND, *indica*; AUS, *aus*; TRJ, *tropical japonica*, TEJ, *temperate japonica*. Arrows on the graph represents admixture events between different rice populations.

# References

Browning SR, Browning BL. 2007. Rapid and accurate haplotype phasing and missing-data inference for whole-genome association studies by use of localized haplotype clustering. *Am J Hum Genet* **81**(5): 1084-1097.

Garris AJ, Tai TH, Coburn J, Kresovich S, McCouch S. 2005. Genetic structure and diversity in *Oryza sativa* L. *Genetics* **169**(3): 1631-1638.

Huang X, Kurata N, Wei X, Wang ZX, Wang A, Zhao Q, Zhao Y, Liu K, Lu H, Li W et al. 2012a. A map of rice genome variation reveals the origin of cultivated rice. *Nature* **490**(7421): 497-501.

Huang X, Zhao Y, Wei X, Li C, Wang A, Zhao Q, Li W, Guo Y, Deng L, Zhu C et al. 2012b. Genome-wide association study of flowering time and grain yield traits in a worldwide collection of rice germplasm. *Nat Genet* **44**(1): 32-39.

Jin J, Huang W, Gao JP, Yang J, Shi M, Zhu MZ, Luo D, Lin HX. 2008. Genetic control of rice plant architecture under domestication. *Nat Genet* **40**(11): 1365-1369.

Kawahara Y, de la Bastide M, Hamilton JP, Kanamori H, McCombie WR, Ouyang S, Schwartz DC, Tanaka T, Wu J, Zhou S et al. 2013. Improvement of the *Oryza sativa* Nipponbare reference genome using next generation sequence and optical map data. *Rice* **6**(1): 4.

Korneliussen TS, Albrechtsen A, Nielsen R. 2014. ANGSD: analysis of next generation sequencing data. *BMC Bioinformatics* **15**(1): 356.

Li C, Zhou A, Sang T. 2006. Rice domestication by reducing shattering. *Science* **311**(5769): 1936-1939.

Li H, Durbin R. 2009. Fast and accurate short read alignment with Burrows-Wheeler transform. *Bioinformatics* **25**(14): 1754-1760.

Li X, Yan W, Agrama H, Hu B, Jia L, Jia M, Jackson A, Moldenhauer K, McClung A, Wu D. 2010. Genotypic and phenotypic characterization of genetic differentiation and diversity in the USDA rice mini-core collection. *Genetica* **138**(11-12): 1221-1230.

Oka HI. 1988. *Origin of Cultivated Rice*. Elsevier, New York.

Paradis E. 2010. pegas: an R package for population genetics with an integrated-modular approach. *Bioinformatics* **26**(3): 419-420.

Pickrell JK, Pritchard JK. 2012. Inference of population splits and mixtures from genome-wide allele frequency data. *PLoS Genet* **8**(11): e1002967.

Stamatakis A. 2006. RAxML-VI-HPC: maximum likelihood-based phylogenetic analyses with thousands of taxa and mixed models. *Bioinformatics* **22**(21): 2688-2690.

Tamura K, Dudley J, Nei M, Kumar S. 2007. MEGA4: Molecular Evolutionary Genetics Analysis (MEGA) software version 4.0. *Mol Biol Evol* **24**(8): 1596-1599.

Wang H, Xu X, Vieira FG, Xiao Y, Li Z, Wang J, Nielsen R, Chu C. 2016. The power of inbreeding: NGS based GWAS of rice reveals convergent evolution during rice domestication. *Mol Plant* **9**(7): 975-985.

Wang M, Yu Y, Haberer G, Marri PR, Fan C, Goicoechea JL, Zuccolo A, Song X, Kudrna D, Ammiraju JS et al. 2014. The genome sequence of African rice (*Oryza glaberrima*) and evidence for independent domestication. *Nat Genet* **46**(9): 982-988.

Xu X, Liu X, Ge S, Jensen JD, Hu F, Li X, Dong Y, Gutenkunst RN, Fang L, Huang L et al. 2012. Resequencing 50 accessions of cultivated and wild rice yields markers for identifying agronomically important genes. *Nat Biotechnol* **30**(1): 105-111.

Zhao K, Tung CW, Eizenga GC, Wright MH, Ali ML, Price AH, Norton GJ, Islam MR, Reynolds A, Mezey J et al. 2011. Genome-wide association mapping reveals a rich genetic architecture of complex traits in *Oryza sativa*. *Nat Commun* **2**: 467.

Zhao K, Wright M, Kimball J, Eizenga G, McClung A, Kovach M, Tyagi W, Ali ML, Tung CW, Reynolds A et al. 2010. Genomic diversity and introgression in *O. sativa* reveal the impact of domestication and breeding on the rice genome. *PLoS One* **5**(5): e10780.
